# Simultaneous measurement of transverse load and temperature using hybrid structured fiber-optic Fabry–Perot interferometer

**DOI:** 10.1038/s41598-017-11218-9

**Published:** 2017-09-06

**Authors:** Yongfeng Wu, Yundong Zhang, Jing Wu, Ping Yuan

**Affiliations:** National Key Laboratory of Tunable Laser Technology, Institute of Opto-Electronics, Harbin Institute of Technology, Harbin, 150080 China

## Abstract

We experimentally demonstrated a novel fiber-optic hybrid structured Fabry–Perot interferometer with special air-cavity for simultaneous measurement of transverse load and temperature. By the linear phase finite impulse response filters, the transverse load sensitivities of the air-cavity and the silica-cavity are 1272.71 pm/N and −53.07 pm/N, respectively, and temperature sensitivities of the air-cavity and silica-cavity are 1.1 pm/°C and 14 pm/°C. Thus, the different sensitivities of silica-cavity and air-cavity to transverse load and temperature indicate that such a structure can be used to simultaneously measure transverse load and temperature.

## Introduction

In recent years, fiber-optic Fabry–Perot interferometer (FPI) has drawn great attention and been used for various physical quantities sensing, such as temperature^1–3^, strain^4–7^, pressure^8–10^, and transverse load^11^ *et al*., due to its advantages of low cost, high sensitivity, ultra-compactness and reliability. During the physical quantity sensing process, temperature fluctuation will introduce extra error. Normally, temperature compensation is added to the sensing system, which make it quite complex. Another way to solve this problem is to realize concurrent sensing of desired physical quantity and temperature. This is not just diminishing cost and complexity of the sensing system but also solving the temperature-induced crossing-sensitivity issue. Therefore, simultaneous measurement of desired physical quantity and temperature has became an important topic in sensing.

Various hybrid structured FPIs have been fabricated for concurrent measurement of temperature and pressure^12^, temperature and refractive index^13^, pressure and temperature^14, 15^, temperature and strain^16^. In 2012, Pevec S. *et al*. proposed and fabricated hybrid structured FPI which consisted of two low-finesse Fabry–Perot resonators integrated into a standard lead-in single mode fiber (SMF) for simultaneous measurement of pressure and temperature^14^. At the same time, in 2014, Pevec S. *et al*. also proposed and fabricated another simultaneous measurement of pressure and temperature sensor based on hybrid structured FPI by chemical etching. In 2014, Zhou A. *et al*. proposed and fabricated hybrid structured FPI by fusion splice between SMF and several electrical arc discharges for simultaneous measurement of strain and temperature. However, these hybrid structured FPIs reported are not suitable for simultaneous measurement of transverse load and temperature for the reason that cavity heights of these structures are not higher than the cladding diameter of the SMF.

In this paper, a novel fiber-optic hybrid structured FPI with special air-cavity that air-cavity height is higher than the cladding diameter of SMF is proposed and experimentally demonstrated for simultaneously measure transverse load and temperature. The hybrid structured FPI can be easily fabricated by fusion splice SMF to silica capillary and then electrical arc discharge melting capillary to become hollow microsphere with special air-cavity, and final fusion splice SMF to hollow microsphere and cleaving to form silica-cavity. The transverse load sensitivity of air-cavity is positive, on the contrary, silica-cavity is negative. In addition, silica-cavity to temperature is more sensitive compared to air-cavity. Therefore, the hybrid structured FPI proposed is appropriate for application to simultaneously measure transverse load and temperature.

## Fabrication and Principle

**Figure 1:**
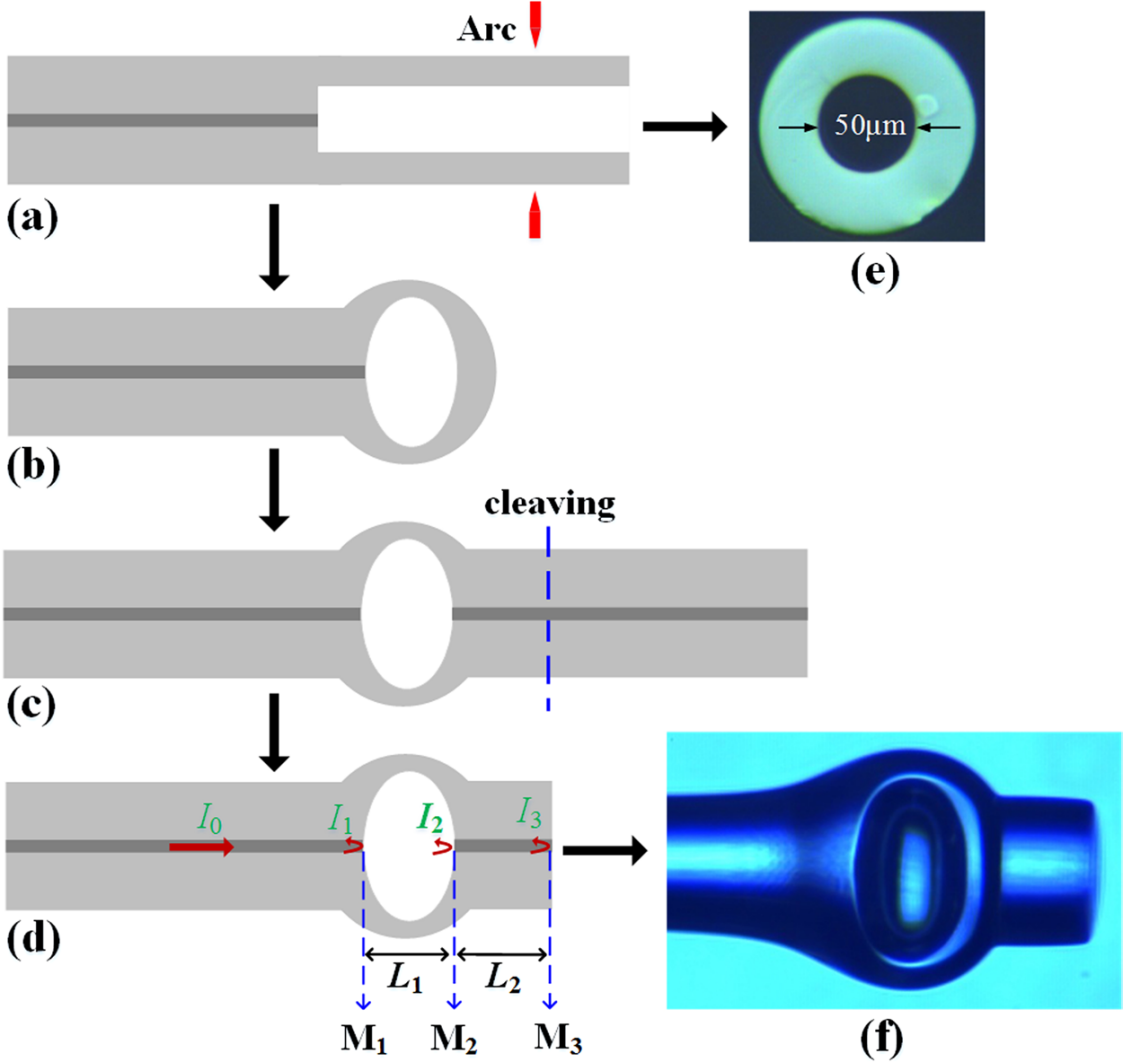
(**a**)–(**d**) Schematic of the fabrication process of hybrid FPI; (**e**) the microscope image of silica capillary cross-section with outer diameter of 125 μm and inner diameter of 50 μm; (**f**) the microscope image of hybrid FPI.

The process of the hybrid structured FPI proposed is shown in Fig. 1(a)–(d). Firstly, a silica capillary with outer diameter of 125 μm and inner diameter of 50 μm as shown in Fig. 1(e) was spliced to SMF by fiber fusion splicer (Fujikura FSM-45PM), as shown in Fig. 1(a). In order to guarantee silica capillary not collapsed during the fusion splice process, the fusion splicer was set to special parameters that arc discharge power was −100 bit, and arc discharge duration time was 400 ms and arc distance from discharge position to fusion splice point was 120 μm. Furthermore, arc of extremely strong (70 bit) and long duration time (2000 ms) discharge deviating about 160 μm from splice point were used to make sure that silica capillary was completely collapsed and cut off to form hollow microsphere with an air-cavity, as shown in Fig. 1(b). Finally, SMF was spliced to the end of the hollow microsphere and SMF was cleaved to become silica-cavity, as shown in Fig. 1(c) and (d). The microscope image of hybrid structured FPI is shown in Fig. 1(f). The air-cavity height and length are respectively 170 μm and 85 μm, and silica-cavity length is 130 μm.

As shown in Fig. 1(d), *I*_1_, *I*_2_, and *I*_3_ are light intensities reflected by reflective surface M_1_, M_2_, and M_3_, respectively; *L*_1_ and *L*_2_ are respectively the air-cavity and silica-cavity length. The intensity of the interference fringes can be written as

1 $$I={I}_{1}+{I}_{2}+{I}_{3}+2\sqrt{{I}_{1}{I}_{2}}\,\cos ({\varphi }_{{\rm{air}}})+2\sqrt{{I}_{2}{I}_{3}}\,\cos ({\varphi }_{{\rm{silica}}})+2\sqrt{{I}_{1}{I}_{3}}\,\cos ({\varphi }_{\mathrm{air} \mbox{-} \mathrm{silica}})$$

where *ϕ*_air_ = 4π*n*_1_ *L*_1_/λ, *ϕ*_silica_ = 4π*n*_2_ *L*_2_/λ, *ϕ*_air-silica_ = *ϕ*_air_ + *ϕ*_silica_, are the phase shifts corresponding to air-cavity, silica-cavity, and the hybrid-cavity, respectively; *n*_1_ and *n*_2_ are respectively refractive indexes of air and SMF; *λ* is the incident light wavelength.

**Figure 2:**
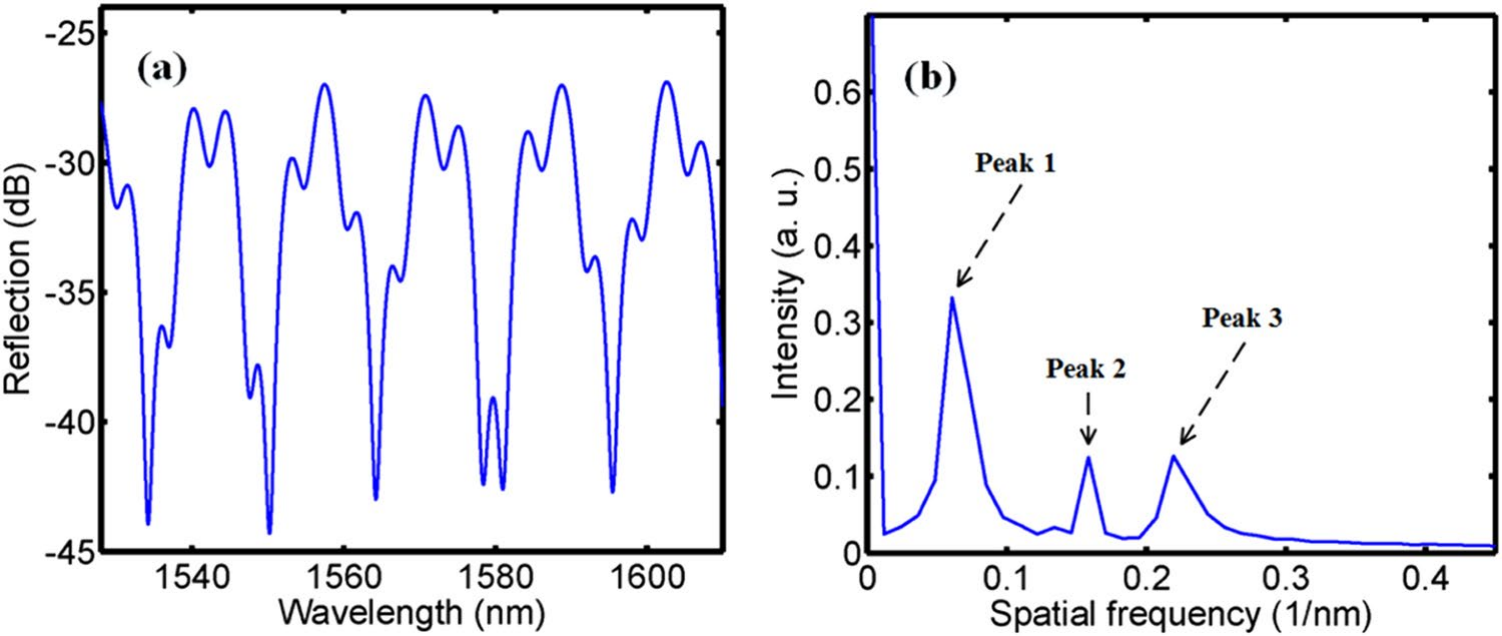
(**a**) Reflection spectrum and (**b**) spatial frequency spectrum.

**Figure 3:**
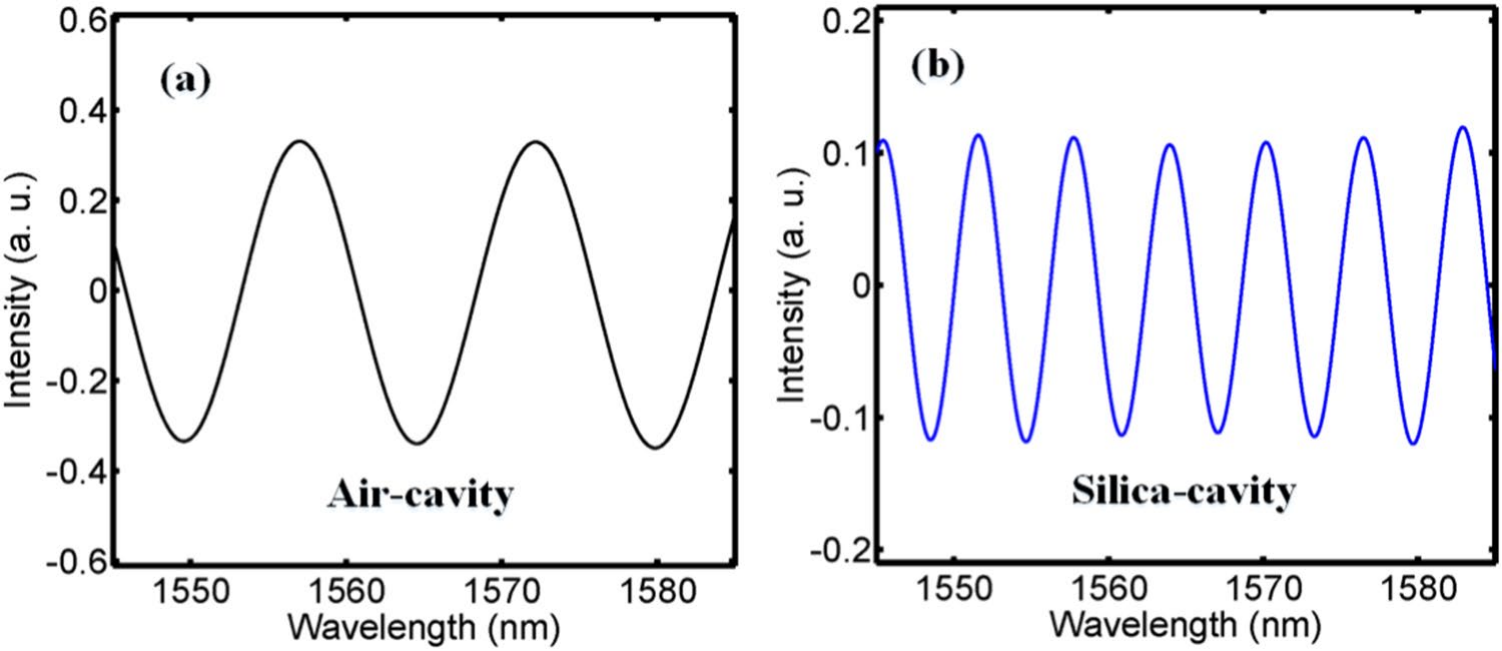
The wavelength spectra after FIR filtering. (**a**) The air-cavity (**b**) the silica-cavity.

The reflection spectrum was observed by a broadband source, 3 dB coupler and an optical spectrum analyzer. The reflection spectrum of the hybrid structured FPI is shown in Fig. 2(a). The spatial frequency spectrum was acquired by fast Fourier transform of the reflection spectrum, as shown in Fig. 2(b). There are three peaks in the spatial frequency spectrum that peak 1, peak 2 and peak 3 are resulted from air-cavity, silica-cavity and hybrid-cavity (air plus silica). The spatial frequency values of peak 1, peak 2 and peak 3 are *f*_1_ = 2*n*_1_ *L*_1_/*λ*_1_ *λ*_2_, *f*_2_ = 2*n*_2_ *L*_2_/*λ*_1_ *λ*_2_ and *f*_3_ = *f*_1_ + *f*_2_, respectively, where *λ*_1_ and *λ*_2_ are two adjacent dips wavelengths of the reflection spectrum. By the linear phase finite impulse response filters, the wavelength spectra of air-cavity and silica-cavity can be extracted from the reflection spectrum, as shown in Fig. 3.

Such a hybrid structured FPI can be used to simultaneously measure transverse load and temperature. The transverse load increasing can cause the air-cavity height to shorten and air-cavity length to lengthen. Thus, spectrum of air-cavity is redshift with the transverse load increasing. At the same time, since phase shift of silica-cavity is reducing with the transverse load increasing, spectrum is blueshift with the transverse load increasing for silica-cavity. In addition, for temperature sensing, air-cavity is only affected by thermal expansion coefficient of silica, and silica-cavity is affected by the thermal expansion coefficient and the thermo-optic coefficient of silica, thus the silica-cavity is more sensitive to temperature than air-cavity. Since the air-cavity and the silica-cavity are different response to transverse load and temperature, this hybrid structured FPI can realize to simultaneously measure transverse load and temperature.

## Experiments and Discussions

**Figure 4:**
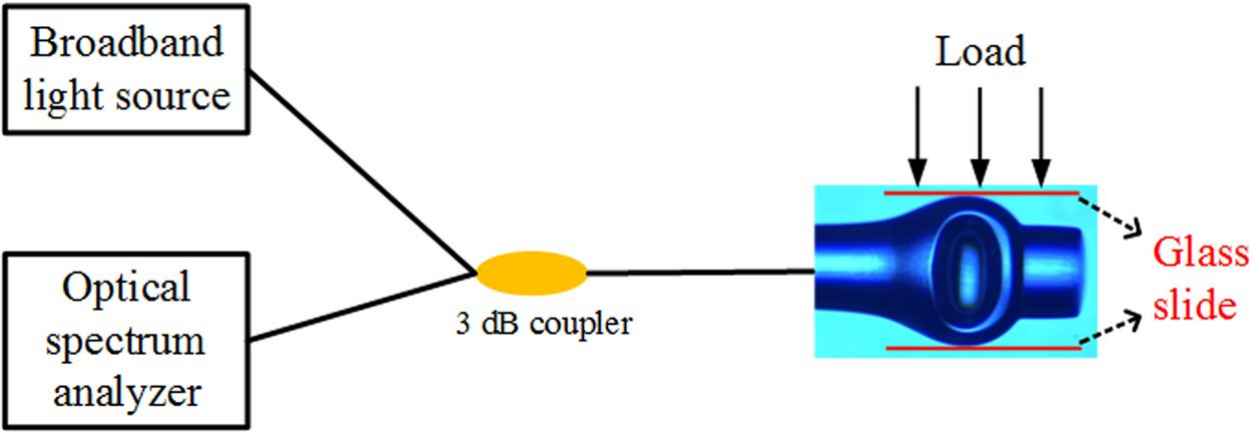
Experimental setup of transverse load measurement.

**Figure 5:**
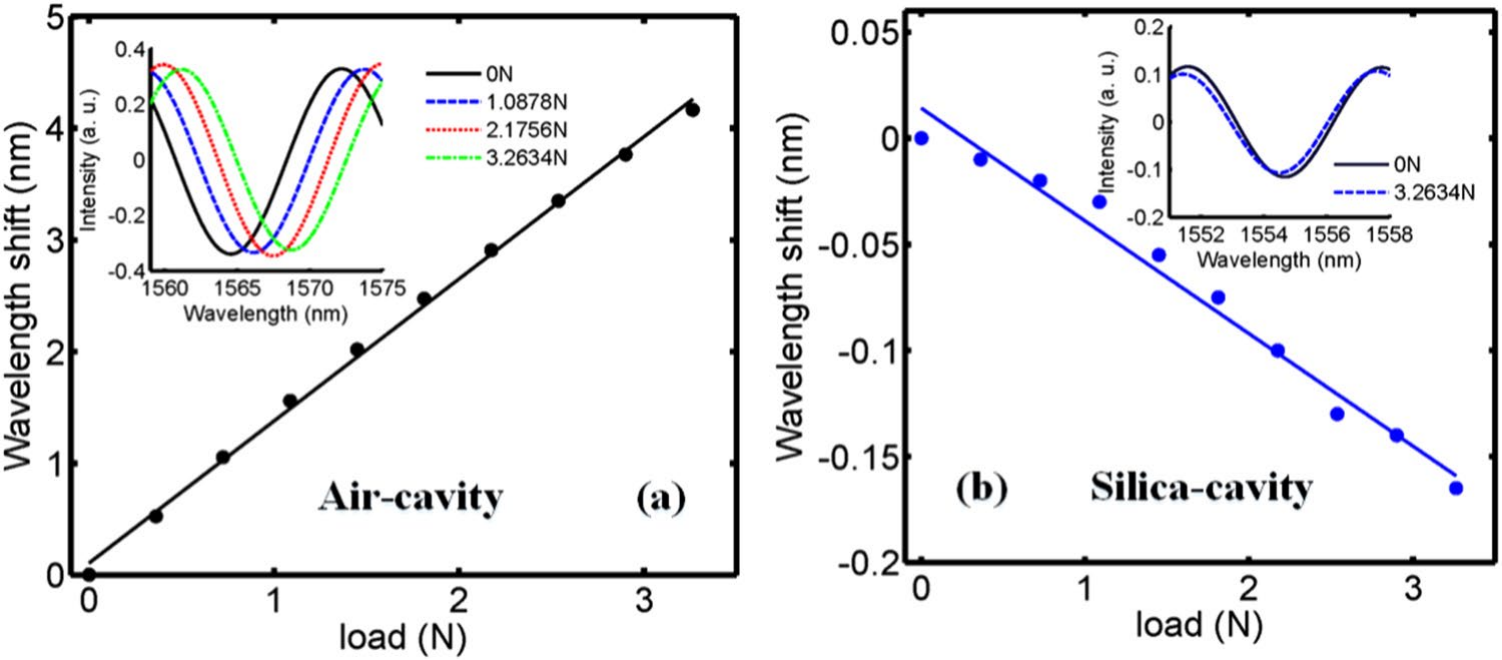
The transverse load response. (**a**) Air-cavity and (**b**) silica-cavity; insets show spectra under different transverse load.

The experimental setup of transverse load for this hybrid structured FPI is shown in Fig. 4. The hybrid structured FPI is horizontally placed between two parallel glass slides in the transverse load measurement. As shown in Fig. 5(a), spectrum of air-cavity has a redshift with the transverse load increasing and the transverse load sensitivity of 1272.71 pm/N is acquired. Moreover, with the increasing of transverse loads, spectrum has a blueshift and its sensitivity to transverse load is −53.07 pm/N for silica-cavity, as shown in Fig. 5(b).

**Figure 6:**
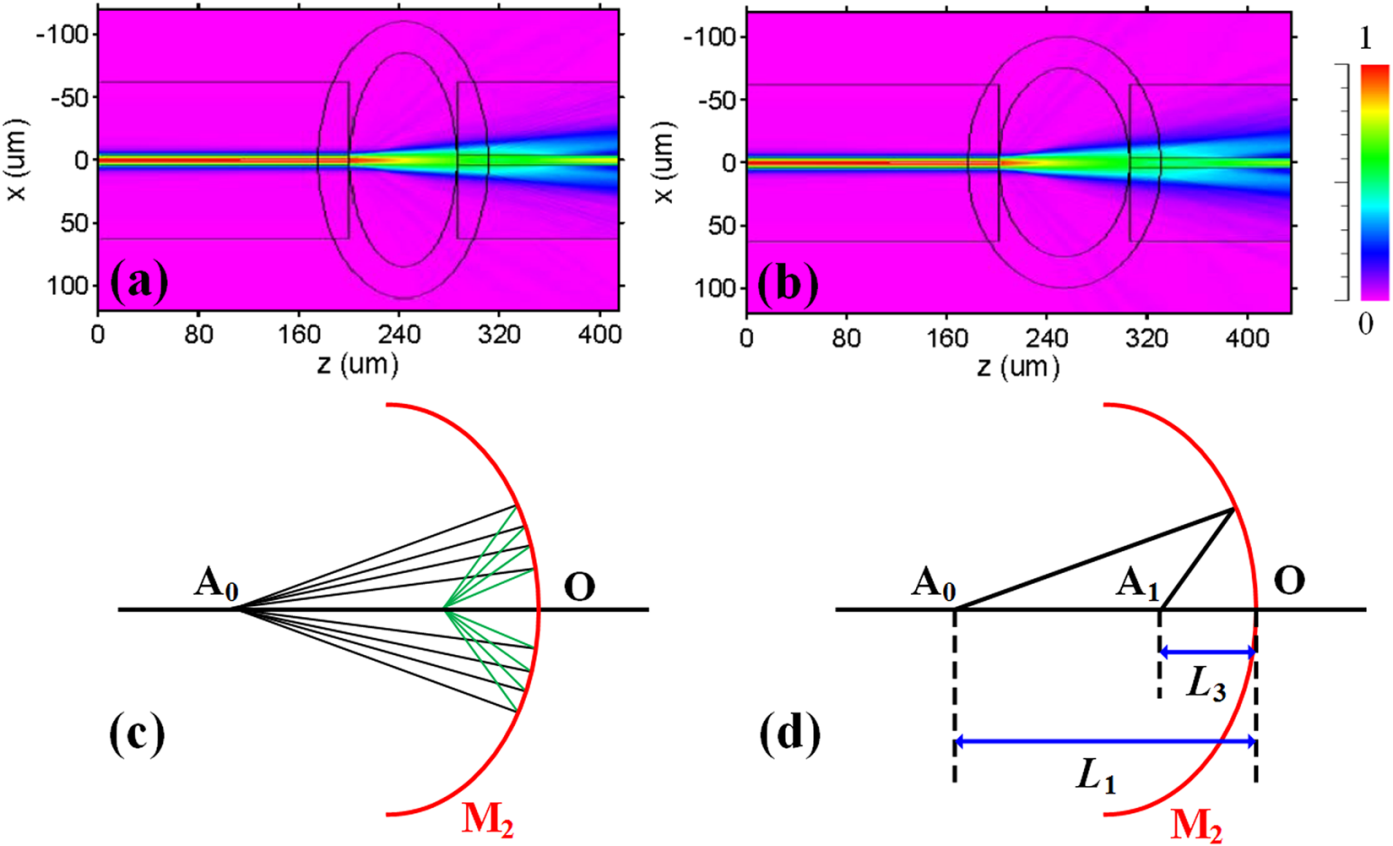
Simulated light propagation in the hybrid structured FPI with silica-cavity length of 130 μm for air-cavities of (**a**) 170 × 85 μm (height × length) and (**b**) 150 × 105 μm. (**c**) Schematic diagram of light reflected by spherical surface converged at convergent point. (**d**) Schematic diagram of spherical reflected light.

Since the transverse load increases, the air-cavity height is shorter and air-cavity length is longer. Therefore, spectrum appears redshift with the transverse load increasing for air-cavity that is in accordance to the experimental results. As shown in Fig. 6(a) and (b), simulated light propagation by beam propagation method in the hybrid structured FPI with silica-cavity length of 130 μm for different shapes of air-cavities, at the input wavelength of 1550 nm, where the z-axis is the light propagation direction. Figure 6(a) and (b) are respectively corresponding to the air-cavities of 170 × 85 μm (height × length) and 150 × 105 μm. From the Fig. 6(a) and (b), light beam reflected by spherical reflector M_2_ can be approximately a near axis optical system. The divergent beam reflected can be converged at convergent point through reflected by spherical, as shown in Fig. 6(c). Therefore, the strongest interference of the two beams reflected by reflective surface M_2_ and M_3_ should be at convergent point. The intensity of the interference fringes for silica-cavity can be accurately written as

2 $$I={I}_{2}+{I}_{3}2\sqrt{{I}_{2}{I}_{3}}\,\cos (\frac{4\pi {n}_{2}{L}_{2}}{\lambda }+\frac{4\pi {n}_{1}{L}_{3}}{\lambda }),$$

where *L*_3_ is the distance between convergent point and reflective surface M_2_. Schematic diagram of spherical reflected light is shown in Fig. 6(d). The relation between object and image of spherical mirror is as follow

3 $$\frac{1}{{L}_{1}}+\frac{1}{{L}_{3}}=\frac{2}{R},$$

where *L*_1_ is distance between beam divergent point and reflective surface M_2_(air-cavity length); *R* is the radius of spherical reflector M_2_. The transverse load makes the air-cavity length (*L*_1_) lengthen as well as air-cavity height shorten so that it causes *R* to become smaller. According to Eq. (3), *L*_3_ is smaller for the reason that *L*_1_ is larger and *R* is smaller with the transverse load increasing. Since phase shift of silica-cavity is smaller as result of smaller *L*_3_ according to Eq. (2), the spectrum is blueshift with the transverse load increasing for silica-cavity. The experimental results are in accordance to the theoretical analysis.

**Figure 7:**
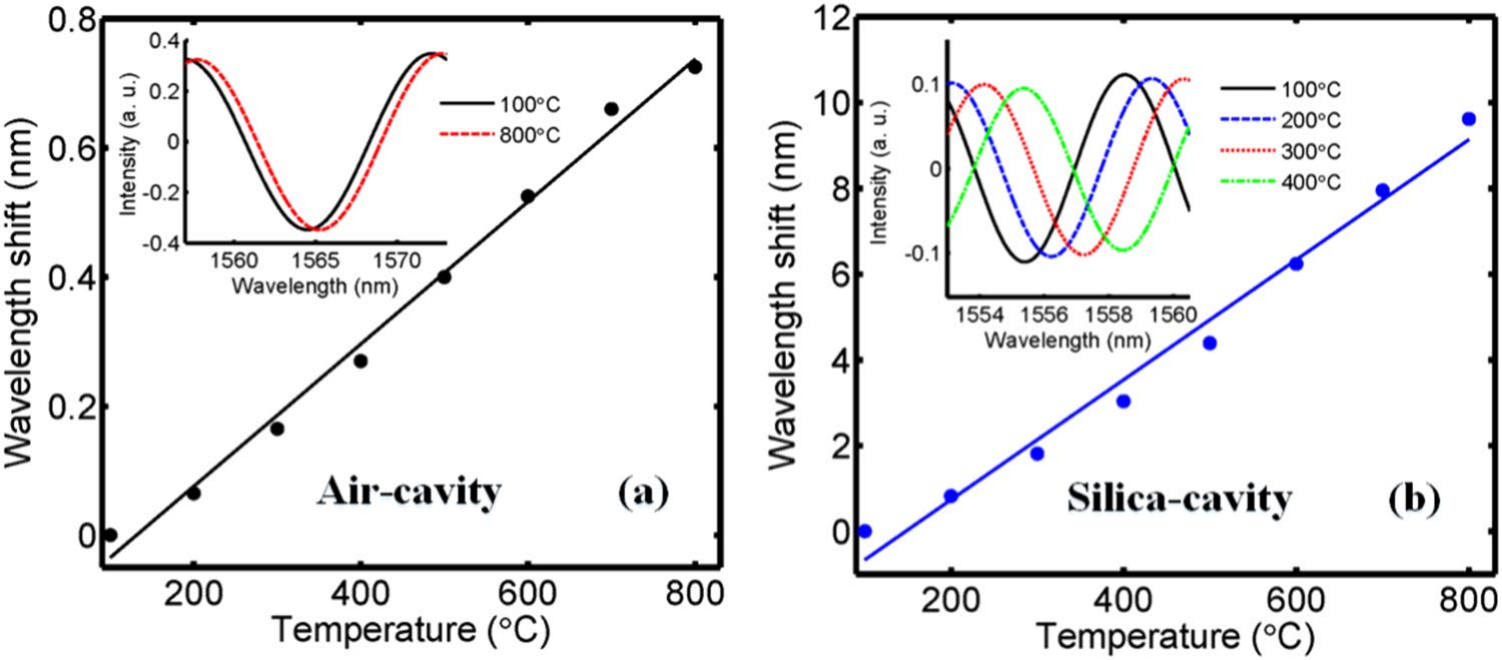
The temperature sensing. (**a**) Air-cavity and (**b**) silica-cavity; insets show spectra under different temperature.

To investigate the temperature response of this structure, the structure is placed in a furnace to raise its temperature from 100 °C to 800 °C with a step of 100 °C. The wavelength shift for the air-cavity and silica-cavity with different temperature are shown in Fig. 7. The temperature sensitivities of the air-cavity and silica-cavity are respectively 1.1 pm/°C and 14 pm/°C. The experimental results show that the silica-cavity is about 10 times more sensitive to temperature than silica-cavity.

The wavelength of dip is respectively *λ*_0_ = 2*n*_1_ *L*_1_/*m* and *λ*_0_ = 2*n*_2_ *L*_2_/*m* for air-cavity and silica-cavity, where *m* is integer. The wavelength shift of air-cavity and silica-cavity to temperature are given by

4 $$\frac{{\rm{\Delta }}\lambda }{{\rm{\Delta }}T}=(\frac{{\rm{\Delta }}L}{{\rm{\Delta }}T\ast \,L}+\frac{{\rm{\Delta }}n}{{\rm{\Delta }}T\ast \,n})\lambda =(\varepsilon +\kappa )\lambda $$

where *ε* = 5.5 × 10^−7^ and *κ* = 1.0 × 10^−5^ are respectively the thermal expansion coefficient and the thermo-optic coefficient for silica^11^. It is obviously that the thermal expansion coefficient only affects air-cavity, however the thermal expansion coefficient and the thermo-optic coefficient affect silica-cavity. The silica-cavity is about 10 times more sensitive to temperature than air-cavity for the reason that the thermo-optic coefficient is over 10 times larger than the thermal expansion coefficient for silica. The experimental results are in accordance to the theoretical analysis.

The experimental results show spectrum has redshift to transverse load for air-cavity whereas spectrum of silica-cavity has blueshift. In addition, the sensitivity of silica-cavity is more over 10 times to temperature than the air-cavity. Due to different response of air-cavity and silica-cavity to transverse load and temperature for this structure, it can realize to simultaneously measure transverse load and temperature. The resolution matrix for concurrent measurement can be expressed as

5 $$[\begin{array}{c}{\rm{\Delta }}N\\ {\rm{\Delta }}T\end{array}]=[\begin{array}{c}{k}_{{\rm{11}}}\\ {k}_{{\rm{21}}}\end{array}{\begin{array}{c}{k}_{{\rm{12}}}\\ {k}_{{\rm{22}}}\end{array}]}^{-1}[\begin{array}{c}{\rm{\Delta }}{\lambda }_{{\rm{air}}}\\ {\rm{\Delta }}{\lambda }_{{\rm{silica}}}\end{array}]$$

where *k*_11_ and *k*_12_ are respectively the transverse load and temperature sensitivity of the air-cavity and *k*_21_ and *k*_22_ are respectively the transverse load and temperature sensitivity of the silica-cavity. In the matrix, Δ*λ*_air_ and Δ*λ*_silica_ represent the wavelength shifts of air-cavity and silica-cavity, respectively; Δ*N* and Δ*T* are respectively variations of transverse load and temperature.

**Figure 8:**
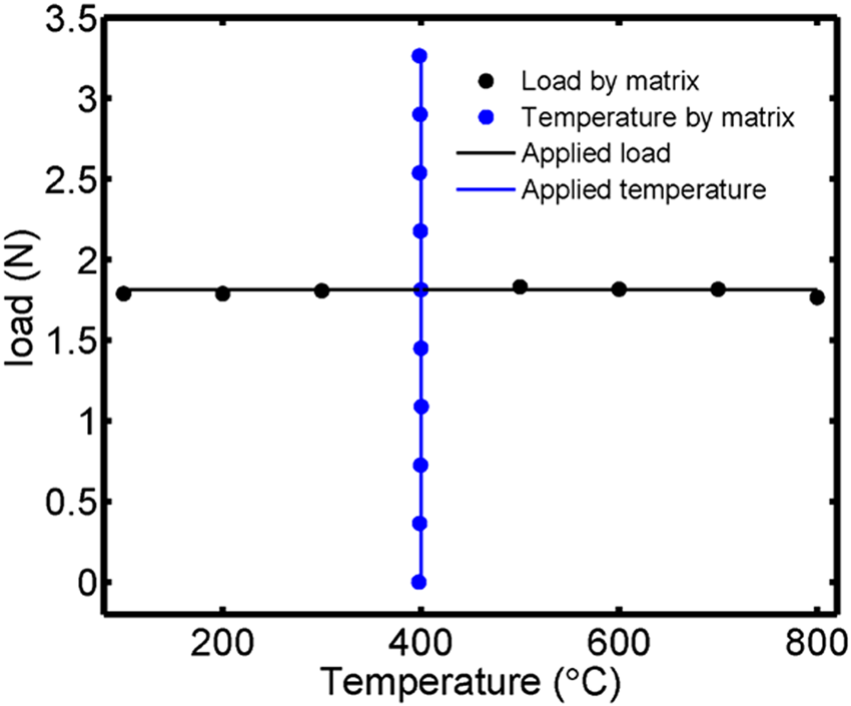
Comparison between applied and matrix method.

The performance of simultaneous measurement of transverse load and temperature for the sensor was estimated by resolution matrix. Choosing 400 °C as reference temperature, Δ*λ*_air_ and Δ*λ*_silica_ obtained transverse load variations in a range of 0–3.2634 N are inputted into resolution matrix to analyze the effects of varying transverse load on temperature measurement. Selecting 1.813 N as reference transverse load, Δ*λ*_air_ and Δ*λ*_silica_ acquired temperature variation from 100 °C to 800 °C are brought into resolution matrix to investigate the effects of changing temperature on transverse load measurement. As shown in Fig. 8, the maximum deviations calculated by resolution matrix are respectively ~0.0489 N and ~2 °C for simultaneous measurement of transverse load and temperature.

## Conclusions

In conclusion, a novel hybrid structured fiber-optic FPI is proposed and experimental demonstrated for simultaneous measurement of transverse load and temperature with the advantages of high sensitivity, low cost and compact, and easy fabrication. Owing to different response of air-cavity and silica-cavity to transverse load and temperature, simultaneous measurement of transverse load and temperature can be easily achieved by a resolution matrix method. Experimental results indicate that such a sensor is suitable for application to concurrently measure transverse load and temperature.
